# The Diagnostic Contribution of SPECT/CT Imaging in the Assessment of Gastrointestinal Bleeding: Especially for Previously Operated Patients

**DOI:** 10.4274/mirt.galenos.2020.24392

**Published:** 2021-02-09

**Authors:** Selin Soyluoğlu, Ülkü Korkmaz, Büşra Özdemir, Gülay Durmuş Altun

**Affiliations:** 1 Trakya University Faculty of Medicine, Department of Nuclear Medicine, Edirne, Turkey

**Keywords:** Gastrointestinal hemorrhage, red blood cell scintigraphy, single photon emission computed tomography/computed tomography

## Abstract

**Objectives:**

Gastrointestinal bleeding (GIB) is a life-threatening problem that requires a multidisciplinary approach for successful treatment. This study aims to emphasize the clinical contribution of single photon emission computed tomography/computed tomography (SPECT/CT) for the diagnosis of acute bleeding.

**Methods:**

All 14 patients referred to the nuclear medicine department in 3 years with suspicion of acute GIB were evaluated retrospectively. Clinical records were analyzed to assess the scintigraphic findings, emphasizing the correlative contribution of the CT portion on SPECT/CT studies.

**Results:**

Five patients were negative on dynamic and static planar images. SPECT/CT was performed in 9 patients who had positive findings on planar imaging. SPECT/CT could identify the same bleeding site originating from the anastomosis in four patients with a history of abdominal surgery. SPECT/CT confirmed bleeding from the cecum in a patient with cervical cancer. SPECT/CT showed the bleeding focus in the bladder neck of a patient with bladder cancer and the bleeding from peritoneal metastases of a patient with gastric cancer. In 1 patient, the right upper quadrant activity accumulation, which may cause false positives, was found to be the gallbladder on SPECT/CT. Delayed images showed the true bleeding focus in the cecum. In 1 patient, suspicious activity accumulation in the midline of the abdomen was found to be due to a previously unknown aortic aneurysm on SPECT/CT.

**Conclusion:**

SPECT/CT imaging is a feasible technique to facilitate image interpretation in patients with GIB. SPECT/CT imaging can guide the surgeon through more accurate localization. Therefore, for proper patient management, SPECT/CT should be applied to detect the bleeding focus, if present, especially in patients who had undergone a previous operation.

## Abstract (tr)

**Amaç:**

Gastrointestinal kanama (GİK), başarılı bir tedavi için multidisipliner yaklaşım gerektiren hayati önemde bir sorundur. Çalışmamızda akut kanama tanısında tek foton emisyonlu bilgisayarlı tomografi/bilgisayarlı tomografinin (SPECT/BT) klinik katkısını vurgulamayı amaçladık.

**Yöntem:**

Üç yıllık bir dönemde akut GİK şüphesi ile nükleer tıp bölümüne başvuran 14 hastanın tümü retrospektif olarak incelendi. Sintigrafik bulguları, SPECT/BT çalışmalarında BT kısmının korelatif katkısına vurgu yaparak değerlendirebilmek için klinik kayıtlar analiz edildi.

**Bulgular:**

Beş hastanın dinamik ve statik görüntüleme bulguları negatifti. Planar görüntülerde pozitif bulguları olan 9 hastaya SPECT/BT yapıldı. SPECT/BT, abdominal cerrahi öyküsü olan dört hastada anastomozdan kaynaklanan kanama yerini tam olarak gösterdi. Serviks kanseri tanılı bir hastada çekumdan kanama gösterildi. SPECT/BT, mesane kanseri olan bir hastanın primer tümörünün arka duvarından kaynaklanan kanama odağını ve mide kanseri olan diğer bir hastanın periton metastazına uyan alanda aktivite birikimini saptadı. Bir hastada sağ üst kadranda yanlış pozitifliğe neden olabilecek aktivite birikimi SPECT/BT’de safra kesesi olarak bulundu ve devam edilen geç görüntülerde çekumda gerçek kanama odağı saptanabildi. Bir hastada, karın orta hatta izlenen şüpheli aktivite birikiminin SPECT/BT’de, daha önceden bilinmeyen aort anevrizmasına bağlı olduğu bulundu.

**Sonuç:**

SPECT/BT görüntüleme, GİK’li hastalarda görüntü yorumlanmasını kolaylaştırmak için kullanılabilecek bir tekniktir. SPECT/BT, tam lokalizasyonun belirlenebilmesi sayesinde cerraha rehberlik edebilir. Bu nedenle, özellikle geçirilmiş operasyon öyküsü bulunan hastalarda kanama odağını tespit etmek için SPECT/BT uygulanmalıdır.

## Introduction

Gastrointestinal bleeding (GIB) is a serious clinical problem with 10% mortality despite advanced diagnostic and treatment methods. Eighty percent of lower GIB stop spontaneously, but 25% of them start to bleed again, and about 10%-15% require emergency surgery. It is essential to find the bleeding site before any intervention. If the location of lower GIB cannot be determined, surgical treatment options, such as left hemicolectomy, blind segmental colectomy, radical subtotal colectomy, or multiple colostomies, may be required to control bleeding. However, despite all these efforts, the source of upper or lower GIB may be due to unpredictable causes (1,2,3,4,5).

Diagnosis is based on history, physical examination, laboratory, endoscopy, selective angiography, and technetium-99m (Tc-99m) labeled red blood cell (RBC) scintigraphy. Compared with angiography, the advantages of the scintigraphic method are non-invasive and can show bleeding at lower rates, such as 0.05-0.1 mL/min compared with 0.5 mL/min for angiography. While endoscopy and angiography often fail to show intermittent bleeding, it is possible to perform imaging of the entire abdomen until the next day with a single radioactive drug dose administered for the scintigraphic method, allowing intermittent and slower bleeding rates to show. Therefore, some authors recommend evaluating patients by scintigraphic methods before endoscopy or angiography (6,7).

GIB scintigraphy can be performed with Tc-99m RBC or Tc-99m sulfur colloid. However, Tc-99m sulfur colloid has a lower sensitivity due to background activity in the reticuloendothelial system and shorter intravascular half-life. Tc-99m RBC allows continuous imaging over many hours with a convenient intravascular half-life.

Tc-99m RBC GIB scintigraphy is routinely started with 30 minutes of dynamic imaging. Continuous monitoring should be performed as far as possible to identify the source of bleeding. If no GIB is detected, a minimum of 60 minutes of initial imaging is recommended.

The correct localization of the bleeding site can be made by identifying the extravasated blood’s initial location and monitoring the blood’s movement from that region in the gut lumen. More images may be required to differentiate small bowel bleeding from large intestinal bleeding. The guideline recommends adding single photon emission computed tomography (SPECT) or SPECT/computed tomography (CT) to the imaging to localize the site precisely (8).

SPECT/CT hybrid devices are imaging systems that allow both SPECT and CT imaging to be performed using the same patient bed in the same system. In this way, both pathophysiological information from SPECT imaging and morphological information from CT can be obtained simultaneously. Anatomic localization of the lesions can be performed more accurately and efficiently. SPECT/CT imaging more useful than SPECT and CT imaging alone by improving localization of abnormal and physiologic findings, providing additional information for interpretation, and ensuring definitive diagnostic certainty (9).

This study aims to determine the contribution of SPECT/CT to the clinic by comparing the results of SPECT/CT fusion images taken in addition to standard planar images in patients referred to our department for Tc-99m RBC scintigraphy for the differential diagnosis of acute GIB.

## Materials and Methods

Patients referred to the nuclear medicine department to identify a bleeding site between January 2017 and January 2020 were evaluated retrospectively. Fourteen patients who underwent Tc-99m RBC scintigraphies between these dates were included in the study.

The modified *in vivo* method was used to label RBCs. A lyophilized pyrophosphate (PYP) kit was prepared by diluting 6 cc saline. The kit was incubated for 10 minutes at room temperature, and patients were injected with 2-3 ccs of PYP intravenously. After 20 minutes, each patient had 15 ccs of blood drawn into a heparin-washed syringe. After adding 20 mCi Tc-99m, the injector was incubated for 20 minutes at room temperature with little shaking. At the end of the incubation period, all injector contents were injected into the patients.

After radioactivity administration, nuclear angiography images were acquired immediately at 1 second per frame for 1 minute, then with dynamic images at 60 seconds per frame for 60 minutes (matrix size, 128x128 pixels). At the end of dynamic imaging, additional static images were taken in posterior and lateral projections. If images were out of focus or there were no suspicious findings in the first evaluation, late static images were taken hourly at 2-6 hours (matrix size, 256x256 pixels). Static imaging was continued until 24 hours in cases evaluated as negative. In the early dynamic and subsequent static images, extra Tc-99m RBC accumulation sites other than the physiological areas and vascular structures were interpreted as positive and continued to delay imaging for accurate localization. SPECT/CT was performed when an abnormal RBC accumulation site was suspected based on the planar imaging findings. All SPECT/CT images were acquired using a hybrid system (GE Healthcare, Optima NM/CT 640). SPECT data were acquired for the region of interest (matrix size, 128x128 pixels, 6° angle steps, 20 s/frame). The acquisition parameters for CT were: 130 keV, pitch 1.0, rotation time 0.6 s, and slice thickness 2.5 mm.

Planar and SPECT/CT images were evaluated by two experienced nuclear medicine specialists. All final judgments were made by consensus.

All case images, reports, and follow-up files were evaluated individually to determine the relative performance of planar and SPECT/CT imaging. The additional contribution of SPECT/CT to detect the presence/absence of bleeding and identify the correct localization during scintigraphic evaluation. Patients were followed up to verify the planar and SPECT/CT results.

This retrospective study was performed in accordance with the ethical concepts of the Declaration of Helsinki, October 2013, and approved by the institutional ethical review board (approval number: 22 April 2019-TUTF-BAEK 2019/185). Informed consent was obtained from participants.

Descriptive statistics were used to describe the demographic characteristics of the patients. No other statistical method was needed.

## Results

A total of fourteen patients, six females and eight males, aged between one and 75 (mean, 54.4±6.4) years, were included in the study.

Five patients were negative on dynamic and static planar images. Since in our department, SPECT/CT is not routinely applied to patients whose planar images are negative, in accordance with the guideline (8), SPECT/CT was not required in these patients. One patient had a colonoscopy diagnosed with chronic colitis. Three patients had endoscopy; their diagnoses were hemorrhagic antral gastritis, erythematous antral gastritis, and chronic gastritis. No further diagnostic study was required because they did not have any evidence of further bleeding, and their clinical findings improved. These patients were considered true negatives.

Nine patients had a suspicious appearance of bleeding on planar images; SPECT/CT was performed on all of them. The clinical and scintigraphic characteristics of all patients are summarized in Table 1.

Three patients had a high probability of bleeding at early planar images. One patient had a history of gastroenteropancreatic-neuroendocrine tumor (GEP-NET), and the other 2 had gastric cancer. They all had a Roux-en-Y surgical procedure. SPECT/CT imaging was performed to enhance the anatomy altered after surgery and the relationship between bleeding and the operation site. In SPECT/CT fusion images, it was evident that the bleeding focus matched the anastomosis area at the operation site. The patient with GEP-NET and one of the gastric cancer patients underwent a second operation to stop bleeding. Surgical intervention was not required for the other patient because of the patient’s low bleeding rate and stable vital findings that did not progress. Follow-up was continued with oncological treatment.

**Figure 1:**
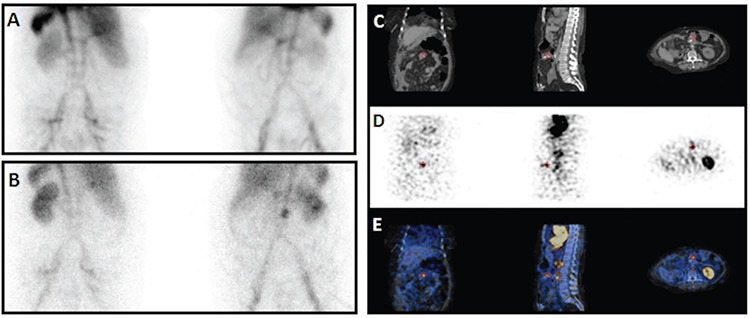
A 73-year-old female with a history of colon cancer, had no signs of bleeding on the 1^st^ day of scintigraphic imaging [(A) posterior and anterior planar images]. At the 24^th^ hour, a focal activity accumulation in the upper quadrant of the abdomen was revealed [(B) posterior and anterior planar images]. SPECT/CT images showed that bleeding focus was at the operating site of colon cancer [(C) CT, (D) SPECT, (E) fused SPECT/CT images in coronal, sagittal, and axial planes, respectively] SPECT/CT: Single photon emission computed tomography/computed tomography

In 1 of the 2 patients whose early images were negative for hemorrhage, in the second-hour study, there was a suspected area in the right lower quadrant. The patient had been treated for cervical cancer with chemotherapy and pelvic radiotherapy. SPECT/CT imaging was performed to identify the location of the bleeding. On SPECT/CT, the hemorrhage was reported to be consistent with the cecum. The anticoagulant drug that the patient was using was discontinued, and an elective colonoscopy was planned. However, as the bleeding findings regressed, it was not needed. The other patient’s first day images showed no signs of bleeding. At the 24^th^ hour, the planar image revealed a focal accumulation area in the abdomen’s upper quadrant. SPECT/CT images of the patient who had a history of operation due to colon cancer revealed that bleeding focus was at the operating site (Figure 1). No further operation was required due to the intermittent feature and low flow rate of bleeding. Oncological treatment was continued.

The moderate activity accumulation area observed in the early images of a patient at the midline of the abdomen’s upper quadrant showed an atypical diffuse distribution on late images. The patient was diagnosed with metastatic gastric cancer and had no operation. SPECT/CT imaging was performed to localize the extravasated activity. Based on the findings of CT images, it was concluded that the activity in the upper abdomen was related to the hypervascular primary tumor area, and the mild diffuse accumulation in the abdomen that was seen on late images was due to bleeding from peritoneal metastases. Paracentesis was performed, and the peritoneal fluid was hemorrhagic. A permanent peritoneal catheter was inserted.

In a patient who had intermittent hematuria and progressive anemia, labeled RBC scintigraphy was performed to exclude any other bleeding foci. While there was no area to suggest bleeding in the abdomen, SPECT/CT showed that the bleeding focus was in the bladder neck. The patient had a diagnosis of ureteral cancer and bladder cancer and a history of transurethral resection of the bladder repeatedly. It was confirmed that hematuria was solely responsible for the patient’s anemia.

**Figure 2:**
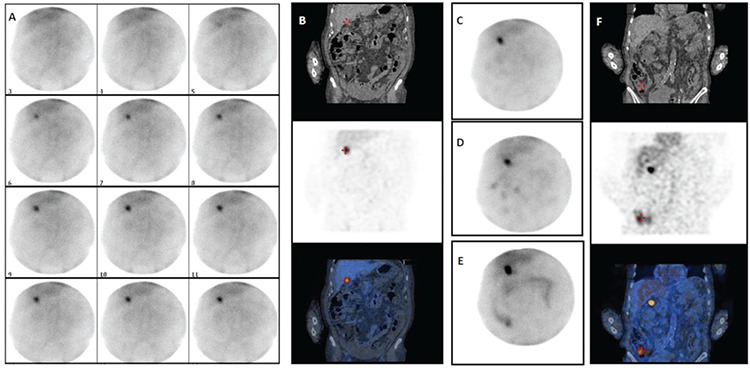
A 72-year-old male with intense activity in the upper right quadrant of the abdomen since the beginning of dynamic images (A). An early SPECT/CT imaging [(B) CT, SPECT, and fused SPECT/CT coronal planes] revealed that this area corresponded to the gallbladder. Therefore, imaging was continued to find the focus of the bleeding. While no additional finding was observed in the first-hour planar images (C), several areas of increased activity appeared in the third (D) and fifth hour (E) planar images. On a second late SPECT/CT, the bleeding was confirmed to originate from the ileocecal valve region [(F) CT, SPECT, and fused SPECT/CT coronal planes] SPECT/CT: Single photon emission computed tomography/computed tomography

In 1 case, early abdomen images showed increased activity in the right upper quadrant of the abdomen. Early SPECT/CT imaging revealed that this activity was due to increased physiological uptake in the gallbladder. Therefore, when imaging was continued to find the focus of the bleeding, in the third hour, several areas of increased activity appeared in the right lower quadrant. In the fifth hour, widespread accumulation of activity was observed in the caecum, ascending colon, transverse colon, and descending colon. A second SPECT/CT scan was performed. On SPECT/CT, the bleeding was confirmed to originate from the ileocecal region. Colonoscopy confirmed submucosal hemorrhages in this region. The anticoagulant drug that the patient was using for a while was discontinued. The bleeding stopped spontaneously. No operation was needed (Figure 2).

**Figure 3:**
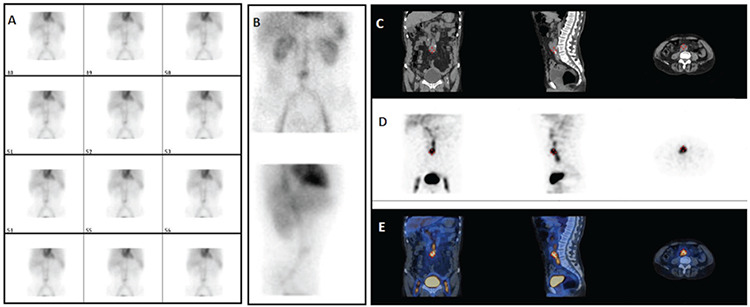
Increased uptake in the midline of the abdomen adjacent to the aorta was noted since the beginning of dynamic images of a 63-year-old male (A). In the late planar images, increased uptake in this area continued, whereas no other area suggested active bleeding in another region [(B) anterior and lateral planar images]. SPECT/CT imaging was performed to interpret this area more accurately with increased uptake. The CT component of SPECT/CT was consistent with the previously unknown aortic aneurysm [(C) CT, (D) SPECT, (E) fused SPECT/CT images in coronal, sagittal, and axial planes] SPECT/CT: Single photon emission computed tomography/computed tomography

Early images of another patient showed increased uptake in the midline of the abdomen adjacent to the aorta. In the late images, increased uptake in this area continued, whereas nothing suggested active bleeding in any other region. SPECT/CT imaging was performed to interpret this area with increased uptake more accurately. In the CT component of SPECT/CT, it was clearly understood that this area was consistent with the previously unknown aortic aneurysm (Figure 3). A bleeding site could not be shown. No further intervention was done as the general condition of the patient improved.

All cases with SPECT/CT images had successful image fusion. Therefore, anatomic localization could be made easily. For cases in which the localization of bleeding could be predicted on planar images, SPECT/CT confirmed the exact location.

## Discussion

GIB scintigraphy is a noninvasive method that can detect bleeding with high sensitivity, localize the bleeding area, and contribute to the predictive ability by showing the approximate bleeding volume (8). However, for some cases, it can be challenging to localize the particular hemorrhage site. Other conventional imaging modalities of the patient may be helpful, but the time interval between them would make it difficult to establish the relationship between bleeding and anatomy. Especially for bleeding that may require surgical procedures, it is more important to determine the exact location of the bleeding. Seven of our patients had previously been treated for various types of cancers. Four of them had a history of abdominal surgery. Knowing the surgically altered anatomy, understanding the characteristics of hypervascular primary tumors and their metastases, and determining the relationship between these areas and activity accumulation areas detected on planar images, can be achieved easily and non-invasively by SPECT/CT. In a study by Schillaci et al. (10), SPECT/CT was able to localize the focus of bleeding in 10 positive cases but non-localizing on planar images. In addition, SPECT/CT changed the results in seven of 19 patients. With the anatomical contribution of CT, SPECT/CT can provide higher overall accuracy than single nuclear imaging.

Four patients in our study had a history of abdominal surgery, and the bleeding site originated at the anastomosis. Three of them were in the jejunojejunal anastomosis region after the Roux-en-Y operation, and the other was in the stapled anastomosis region after colorectal surgery. GIB after GI operations and stapled anastomosis is a rare complication (11,12). SPECT/CT can be essential in operated patients to enhance the understanding of the anatomy altered after surgery and the relationship between bleeding and the operation site (13).

Although nuclear medicine bleeding scintigraphy is mostly used to establish the location of GIB, it is also possible to detect bleeding areas outside of the GI tract. We had 2 examinations, in which the bleeding sites were located outside of the GI tube. One involved the hemorrhage of peritoneal metastases of gastric cancer, and the other involved the bleeding focus in the bladder neck. In cases of suspicious bleeding in patients with a history of trauma or a predisposition to bleeding, Tc-99m RBC imaging may have a role in determining the presence and location of active bleeding in non-GI areas. The reason for this is that Tc-99m RBC imaging has the advantage of imaging for up to 24-hr postinjection and the ability to screen the entire body with a single drug dose. Gonzalez et al. (14) presented three cases of examples as labeled RBC scintigraphy showed the active hemorrhage areas outside of the GI system. Scintigraphy of a patient who had fallen down a flight of stairs two weeks ago and had severe anemia showing increased activity consistent with active bleeding in the chest wall. In another patient with a history of several falls, scintigraphy showed a large, cold defect consistent with a hematoma in his mid-thigh and multiple foci of increased uptake consistent with active bleeding areas around it. Otomi et al. (15) reported 2 patients with bleeding located outside of the GI system, in a study of 20 patients, one massive subcutaneous lumbar hematoma, and one intraperitoneal rupture of a left gastric artery aneurysm. There are several studies in which the bleeding areas have been successfully identified in different parts of the body, such as the extremities, joints, lung, mesenteric region, breast, thyroid, and occult pericardial hemorrhage immediately after open-heart surgery (14,16,17,18). SPECT/CT will also be very beneficial when evaluating such cases.

One particular patient group for whom scintigraphy is even more critical for its non-invasive screening capability of active bleeding foci in the whole body are those with hemophilia, other coagulation disorders, and receiving anticoagulant therapy. In these patients, where invasive techniques are undesirable, any bleeding area’s location and activity become crucial regarding an emergency intervention (19). Park et al. (20) reported that the incidence of GIB was 12.6% (28 of 222 patients) in adult patients with severe aplastic anemia, and in 34.4% (11 patients) of them, the bleeding site was unknown. Even differential diagnosis of chronic arthropathy and acutely bleeding joints can be performed safely in hemophilic arthropathy, which will develop in 50% of patients with hemophilia (21). We had no patients with hemophilia but had 2 patients who were receiving anticoagulant therapy. SPECT/CT could detect the intermittent- and low-volume bleeding sites non-invasively. After positive scintigraphy results, anticoagulant drugs that the patients were using for a while were interrupted.

According to the Society of Nuclear Medicine and Molecular Imaging guideline, it is recommended to start imaging with dynamic nuclear angiography images at a rate of 1-3 seconds per frame for 1 minute (8). Then, dynamic imaging should be continued with a maximum rate of 60 seconds per frame. Imaging should be continued, if possible, for at least one hour until the bleeding source is detected. Due to this, nuclear angiography and subsequent early dynamic images, vascular anatomy, anomalies, and malformations can be easily exposed, and false-positive results can be prevented. For example, in 1 of our patients, increased uptake in the abdomen’s midline adjacent to the aorta was observed since the angiography phase. No other area was found to suggest active bleeding. SPECT/CT imaging was performed to interpret this area of increased uptake in the midline more accurately. In the CT component of SPECT/CT, it was clearly understood that this field was consistent with the previously unknown aortic aneurysm. An abdominal aortic aneurysm is the most common aortic pathology. It is mostly asymptomatic and found incidentally.

Aneurysm rupture is a medical emergency, and surgical intervention is recommended for all symptomatic aneurysms and asymptomatic aneurysms greater than 5.5 cm in diameter. It is fatal in roughly 80% of cases if not treated immediately (22). With Tc-99m RBC scintigraphy, an aortic aneurysm could be diagnosed incidentally. SPECT/CT may contribute by confirming this finding, determining its clinical significance, and whether it requires immediate intervention. In a case presented by Duarte et al. (23), a patient had a Tc-99m RBC scintigraphy to identify GIB, not a GIB site, but a persistent radioactive accumulation seen as a part of the aorta. Because bleeding stopped without intervention, their patient refused the further examination. However, he had a ruptured aortic aneurysm after 17 months. When such previously unknown abnormalities are detected, the addition of SPECT/CT to the standard imaging protocol might be a life-saving contribution to the patient’s clinic and follow-up.

Although not included in our clinical series, many other vascular abnormalities other than aortic aneurysm may be incidentally detected on GIB scintigraphy studies, such as hemangiomas, great vessel tortuosity, varices, arteriovenous malformations, and aortaenteric fistulas (24,25,26,27). Clinicians should be aware that these vascular anomalies may cause false-positive results for GIB. Chen and Brown (28) reported a patient with ileal varices that led to a false-positive interpretation of GIB scintigraphy. Ileal varices in the right lower quadrant filling from the superior mesenteric and ileocolic veins of a patient with previously unknown cirrhosis simulate a GIB pattern. They emphasized that it would have been better if a SPECT/CT was done to determine the activity’s exact location.

When evaluating Tc-99m RBC scintigraphy, activity accumulation in an area outside the normal distribution in the abdominal and pelvic regions is considered positive. However, if the patient’s structural anomalies and other diseases are unknown, false-positive results may occur when the bleeding is interpreted. One of our patients had increased activity in the right upper quadrant of the abdomen on early static images, which was suspicious of bleeding in this region. However, this increased activity did not show any movement, and the intensity of the activity did not change much over time. SPECT/CT revealed that this increased activity belonged to the increased physiological gallbladder uptake. Therefore, when imaging continues to find the true focus of bleeding on late images, additional increased activity areas of the right abdomen were observed from the third hour onwards. In a late second SPECT/CT, activity was concentrated in the ileocecal region, and it was concluded that the bleeding originated here. During Tc-99m RBC scintigraphy, gallbladder visualization is not a common finding but has been reported in the literature. The mechanism of increased uptake in the gallbladder is not well known, but the most common features are renal insufficiency, anemia, and multiple blood transfusions. Our patient had a history of chronic renal failure. As far as we can tell from the literature, there is no typical gallbladder uptake pattern.

In Wang et al.’s (29) case report, no suspicious focus was detected in the first and fifth hours of static images of a 50-year-old patient with chronic renal failure and resultant severe anemia. On the twenty-second hour images, they found suspicious and increased activity at the liver’s inferior border on static images. The subsequently acquired SPECT/CT images located the activity in the gallbladder (29). In Kumar et al.’s (30) case, an abnormal focal uptake in the right hypochondrium was detected on second-hour static images of a 16-year-old boy known for chronic glomerulonephritis and had a history of renal transplantation. Subsequently, SPECT/CT imaging located the uptake in the gallbladder. In our patient, increased uptake in the right upper quadrant compatible with gallbladder fossa was noticed from the beginning of early dynamic images. The literature contains reports demonstrating visualization of the gallbladder at different hours and different intensities during Tc-99m RBC scintigraphy (31,32,33). SPECT/CT can verify that this increased uptake belongs to the gallbladder. Therefore, it helps to avoid false positivity and guides to continue imaging to find the true focus of bleeding.

SPECT/CT can also facilitate the differentiation of other conditions identified as pitfalls in GIB reporting. Physiological genital activities, such as physiological penile activity and endometrial proliferation in the ovulatory cycle, can be mistaken for the bleeding site (34,35). Kidney activity may be confused, especially in unknown abnormalities, such as ectopic and horseshoe kidneys (36,37). Even if it is known, SPECT/CT will make it much easier to make a differentiation. Splenic pathologies, such as the accessory spleen, splenius, and splenic infarct, may also mimic GIB (38,39,40).

Studies showed that SPECT/CT scan could better determine the bleeding site when it cannot be well localized, or indeterminate on planar images, or differentiate physiological causes from pathological activity. In a study, SPECT/CT could indicate a localization in all 10 patients whose location could not be determined by planar imaging. SPECT/CT showed the accurate bleeding focus verified by other modalities and surgery in 12 of 13 patients. In 10 patients where planar imaging localized the bleeding focus, SPECT/CT confirmed seven foci while correcting three localizations (10). In addition to its contribution to detecting and confirming the localization of bleeding, SPECT/CT can more accurately predict the length of the bleeding area and help decide which endoscopic approach to use for evaluation (41).

### Study Limitations

There were some limitations to our study. First, the study was a retrospective, single-institution study with a limited sample size. Similar studies were published before by Schillaci et al. (10) and Otomi et al. (15), as we referenced. This study’s proposed novelty was that SPECT/CT afforded the added benefit of localizing the bleeding, especially in patients with a history of previous operation or cancer, as they made up most of our patient group. We also aimed to demonstrate that there may be a wide variety of causes for false positives that can be quickly resolved with SPECT/CT. This limited number of patients made a significant contribution with SPECT/CT. However, the results of a more extensive series of studies will provide more reliable information about the true value of this contribution.

## Conclusion

Tc-99m RBC scintigraphy is an easily applicable diagnostic test that can show the focus of bleeding, more sensitively than any other technique, even at low bleeding rates or intermittent bleeding.

In addition, if SPECT/CT imaging is added, it can provide information about the etiology of the bleeding site and identify additional anomalies that can cause false positives. Furthermore, SPECT/CT can quickly analyze altered anatomy and the relationship between bleeding site-primary tumor in cancer patients and bleeding site-operation site in previously operated patients. SPECT/CT imaging can guide the surgeon for more accurate localization. Therefore, for proper patient management, SPECT/CT should be applied to detect the bleeding focus, if present.

## Figures and Tables

**Table 1:**
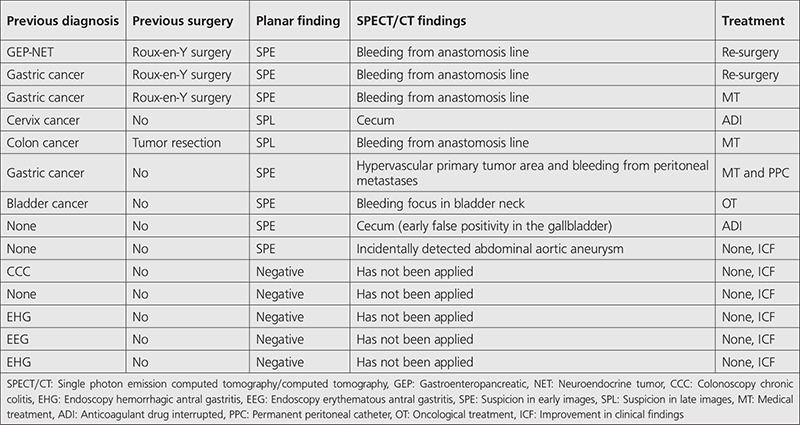
Clinical and scintigraphic characteristics of patients
